# Differential expression of endogenous plant cell wall degrading enzyme genes in the stick insect (Phasmatodea) midgut

**DOI:** 10.1186/1471-2164-15-917

**Published:** 2014-10-21

**Authors:** Matan Shelomi, W Cameron Jasper, Joel Atallah, Lynn S Kimsey, Brian R Johnson

**Affiliations:** Department of Entomology and Nematology, University of California-Davis, Davis, CA 95616 USA; Department of Entomology, Max Planck Institute for Chemical Ecology, 07745 Jena, Germany

## Abstract

**Background:**

Stick and leaf insects (Phasmatodea) are an exclusively leaf-feeding order of insects with no record of omnivory, unlike other “herbivorous” Polyneoptera. They represent an ideal system for investigating the adaptations necessary for obligate folivory, including plant cell wall degrading enzymes (PCWDEs). However, their physiology and internal anatomy is poorly understood, with limited genomic resources available.

**Results:**

We *de novo* assembled transcriptomes for the anterior and posterior midguts of six diverse Phasmatodea species, with RNA-Seq on one exemplar species, *Peruphasma schultei*. The latter’s assembly yielded >100,000 transcripts, with over 4000 transcripts uniquely or more highly expressed in specific midgut sections. Two to three dozen PCWDE encoding gene families, including cellulases and pectinases, were differentially expressed in the anterior midgut. These genes were also found in genomic DNA from phasmid brain tissue, suggesting endogenous production. Sequence alignments revealed catalytic sites on most PCWDE transcripts. While most phasmid PCWDE genes showed homology with those of other insects, the pectinases were homologous to bacterial genes.

**Conclusions:**

We identified a large and diverse PCWDE repertoire endogenous to the phasmids. If these expressed genes are translated into active enzymes, then phasmids can theoretically break plant cell walls into their monomer components independently of microbial symbionts. The differential gene expression between the two midgut sections provides the first molecular hints as to their function in living phasmids. Our work expands the resources available for industrial applications of animal-derived PCWDEs, and facilitates evolutionary analysis of lower Polyneopteran digestive enzymes, including the pectinases whose origin in Phasmatodea may have been a horizontal transfer event from bacteria.

**Electronic supplementary material:**

The online version of this article (doi:10.1186/1471-2164-15-917) contains supplementary material, which is available to authorized users.

## Background

Whole transcriptome shotgun sequencing, or RNA-Seq, is a high-throughput, next-generation sequencing tool that can efficiently identify tens of thousands of functional genes in an organism or specific tissue at a given time [1, 2]. This deep sequencing makes it a more attractive tool than microarrays for organisms lacking reference genomes, facilitating *de novo* transcriptome assembly [3–5]. Its high coverage is desirable when profiling transcripts in tissues of unknown function, enabling researchers to generate and/or test multiple hypotheses at once (eg: [6, 7]), and in organisms potentially harboring symbiotic organisms that may or may not produce the transcripts of interest (eg: [8–10]), as RNA-Seq can simultaneously identify genes from microbes and their vectors/hosts.

Such a combination of low genome resource availability and enigmatic physiology exists in the stick and leaf insects (order Phasmatodea), or phasmids. Though common in the pre-molecular biology era through the Laboratory Stick Insect, *Carausius morosus* [11], phasmid research today is relatively limited. Few phasmids are pests of agricultural crops [11, 12], though they reach plague-like abundances in temperate forests [13, 14] and *C. morosus* is an invasive pest in several countries [15, 16]. All life stages of all species within the order feed exclusively on leaves [17]. This obligate folivory is relatively rare: Among insects it is known only from leaf beetles (Coleoptera: Chrysomelidae), while more basal “herbivores” such as grasshoppers and crickets (Orthoptera) will quite readily scavenge vertebrate meat, engage in cannibalism, and even hunt and kill other insects [18, 19]. Thus phasmids are an ideal system for studying the evolution of herbivory in the lower Polyneoptera.

Folivorous organisms benefit greatly from plant cell wall degrading enzymes (PCWDEs), a group that includes cellulases, hemicellulases, lignases, pectinases, and xylanases [17]. Once thought to be limited to microbes, endogenous (symbiont-independent) PCWDE production has since been found throughout the Animalia. In particular, cellulase (beta-1,4-endoglucanase; Enzyme Commission: 3.2.1.4) genes from the Glycoside Hydrolase family 9 (GH9) are now believed to have existed in the ancestor of all Metazoan life [20, 21] as opposed to having been repeatedly acquired from microbes via horizontal gene transfer, as is thought to be origin of GH45 and GH48 cellulases in beetles [22, 23]. Among insects, endogenous cellulases have been found in lower and higher termites, cockroaches, crickets, beetles [21–24], a firebrat [25], a springtail [26], and, recently, the phasmids. High cellulase activity in the anterior midguts of two phasmid species, *Eurycantha calcarata* and *Entoria okinawaensis*, was detected, the responsible proteins isolated, and the genes encoding them sequenced. Sequence homology and antigency against an insect cellulase anti-serum supported an endogenous, Insectan origin for the enzymes [27]. Such a process is slow and predicated on the translation of PCWDE genes into enzymes active against laboratory substrates like carboxymethylcellulose or crystalline cellulose, whose specificity and selectivity are imperfect [28, 29].

Whether or not phasmids contain other PCWDEs, such as the cellobiases (a type of beta-glucosidase; EC:3.2.1.21) that convert the products of cellulase into glucose monomers [24] or the pectinases (polygalacturonases; EC:3.2.1.15) that hydrolyze pectin into galacturonic acid monomers [17], was unknown. Presence of such active enzymes could explain the obligate folivory of the Phasmatodea and be a key factor in the order’s evolution [30], which is itself a puzzle as the sister order to the Phasmatodea is highly debated [31, 32]. Microbiological assays of the phasmid gut suggest digestion in the order is symbiont-independent [33], so any phasmid PCWDE genes are likely endogenous, yet whether the genes show homology to insect or microbe genes depends on their own evolutionary origin [23, 30]. Complicating the issue is the relative lack of genetic resources for phasmids or their most closely-related orders: Orthoptera [34], Embioptera [35], and Notoptera/Xenonomia [36]. Lastly, even if phasmids have PCWDE genes, their expression is not a guarantee, nor are they necessarily expressed in the gut region where they are most active. The phasmid midgut is physically differentiated into two sections: an anterior midgut (AMG) marked by circular pleating and folding, and a posterior midgut (PMG) studded irregularly by hollow bulbs with filamentous tubules called the “appendices of the midgut” that open into the midgut lumen [37]. The appendices may have an excretory or secretory function, and the possibility exists that they produce digestive enzymes that are carried forward into the AMG via countercurrent flow [38]. In the face of all these unknowns, next-generation sequencing is the best resource for answering questions of phasmid digestive physiology efficiently and effectively.

Here we used *de novo* transcriptome assembly to identify the genes expressed in the midguts of six species of Phasmatodea from four families, while greatly increasing the publicly available genetic resources for the order. We also used RNA-Seq on one exemplar species, *Peruphasma schultei* (Pseudophasmatidae) to quantitatively compare transcript expression between the AMG and PMG, and produced a genomic DNA library from the symbiont-free phasmid brain to confirm that identified transcripts were encoded by the insect itself. Our main goal was to identify the production organ of the Phasmatodea endogenous cellulase, while simultaneously creating an inventory of expressed PCWDE and other digestive genes in phasmids and generating hypotheses on their evolutionary origins and the putative functions of the midgut sections. This study serves as a necessary preliminary for more targeted molecular work. More broadly, our transcriptomes are useful for evolutionary analyses of non-cellulase PCWDEs in insects and identifying potential genes with biotechnological applications such as in processing biofuel feedstock or improving its rheology [39, 40].

## Methods

### Insects and microscopy

Insects used were *Peruphasma schultei* (Pseudophasmatidae), *Sipyloidea sipylus* (Diapheromeridae), *Aretaon asperrimus* (Heteropterygidae), and *Extatosoma tiaratum, Medauroidea extradentata*, and *Ramulus artemis* (Phasmatidae) cultured at room temperature in the Bohart Museum of Entomology, University of California, Davis. Phasmids were fed an *ad libitum* diet of privet (*Ligustrum* sp*.*) for *Peruphasma*, *Eucalyptus* for *Extatosoma*, and *Rosa* sp. for the others.

### Library prep and sequencing

The RNA-Seq study of *Peruphasma schultei* made use of three biological replicates for both the anterior and the posterior midguts (AMG and PMG respectively). For each replicate, the guts of five fed, surface-sterilized, adult, male and female phasmids were removed under sterile conditions and emptied of their contents in several washes of 70% ethanol. Then the anterior and posterior sections were separately pooled and homogenized in TRIzol® Reagent. RNA was extracted according the Trizol-Plus protocol, which includes an on-column DNAase digestion step. Total RNA quality (and subsequent library quality) was checked with the Bioanalyzer 2100. Libraries were made using the Illumina TruSeq v2 kit according to the manufacturer’s instructions.

Hundred base pair paired-end sequencing was performed on the HiSeq 2000 and the raw data uploaded to the NCBI SRA Database [GenBank:SRP030474]. For quality control, low quality bases and adapter contamination were removed with the fastx toolkit [41] and the cutadapt software packages [42]. FastQC [43] was used to check the final quality of reads prior to *de novo* assembly. The number of reads generated for each biological replicate is shown in Table 1.

**Table 1:** **Total reads and trinity results for each transcriptomic or genomic library**

***de novo***stick insect transcriptome assemblies	Reads	Total trinity transcripts (isotigs)	Total trinity components (isogroups)	Contig N50
*Peruphasma schultei*		135622	99469	1669
Anterior midgut 1	15,578,606			
Anterior midgut 2	17,004,583			
Anterior midgut 3	20,651,269			
Total:	53,234,458			
Posterior midgut 1	17,664,733			
Posterior midgut 2	22,326,598			
Posterior midgut 3	21,932,697			
Total:	61,924,028			
*Aretaon asperrimus*		142181	110688	3188
Anterior midgut	57,859,873			
Posterior midgut	41,709,281			
*Extatosoma tiaratum*		163928	117927	1878
Anterior midgut	67,177,740			
Posterior midgut	59,190,949			
*Medauroidea extradentata*		130080	99465	2246
Anterior midgut	54,198,129			
Posterior midgut	49,043,590			
*Ramulus artemis*		169555	92260	2007
Anterior midgut	55,689,810			
Posterior midgut	59,684,645			
*Sipyloidea sipylus*		114125	72103	1257
Anterior midgut	47,511,044			
**Genomic** ***P. schultei*** **reads (brain tissue)**	46,868,237			

Total number of transcripts and components based on results of the Trinity assembler [3] with default parameters. N50 statistic is a nucleotide length.

For gut transcriptomes of the other five species, the same method was used as for *P. schultei* with a few changes. For each species, only one biological replicate was produced for both the anterior and the posterior midgut. This library was made of pooled midguts (all females for *E. tiaratum, M. extradentata, R. artemis,* and *S. sipylus*, and a mixture of males and females for *A. asperrimus*). RNA was successfully extracted for all tissues with the exception of the *S. sipylus* PMG, for which the extraction failed and for which no additional specimens could be obtained. RNA-extraction and quality control were as for *Peruphasma*, but libraries were made using the NEBNext® Ultra™ RNA Library Prep Kit for Illumina kit according to the manufacturer’s instructions. Sequencing was on an Illumina HiSeq 2000, and the data uploaded to the NCBI SRA Database [GenBank:SRP038202]. The numbers of reads produced for each sample are shown in Table 1.

### *de novo*transcriptome assembly

The Trinity assembler with the default parameters was used to generate *de novo* transcriptomes for all species using quality controlled reads [3]. TopHat (v2.04) was used for aligning reads to the transcriptome [44]. HTSeq [45] was used to quantify the number of reads aligning to each transcript. Gene and isoform abundances and expression levels from the *P. schultei* RNA-Seq data were quantified using RSEM (RNA-Seq by Expectation Maximization) [46]. This program was chosen over other programs as it does not rely on reference genomes, of which there are none for the Phasmatodea. For the *P.* schultei RNA-Seq, Trinity assembly yielded 135,622 transcripts (N50 contig length=1669). Differentially expressed genes were identified using EBSeq, an R package that compares isoform expression across two or more biological conditions, in this case AMG and PMG, using a Bayesian heirarchical model [47]. Differentially expressed genes (DEGs) were those with an adjusted p-value <0.05.

### Transcriptome annotation and PCWDE identification

**Figure 1:**
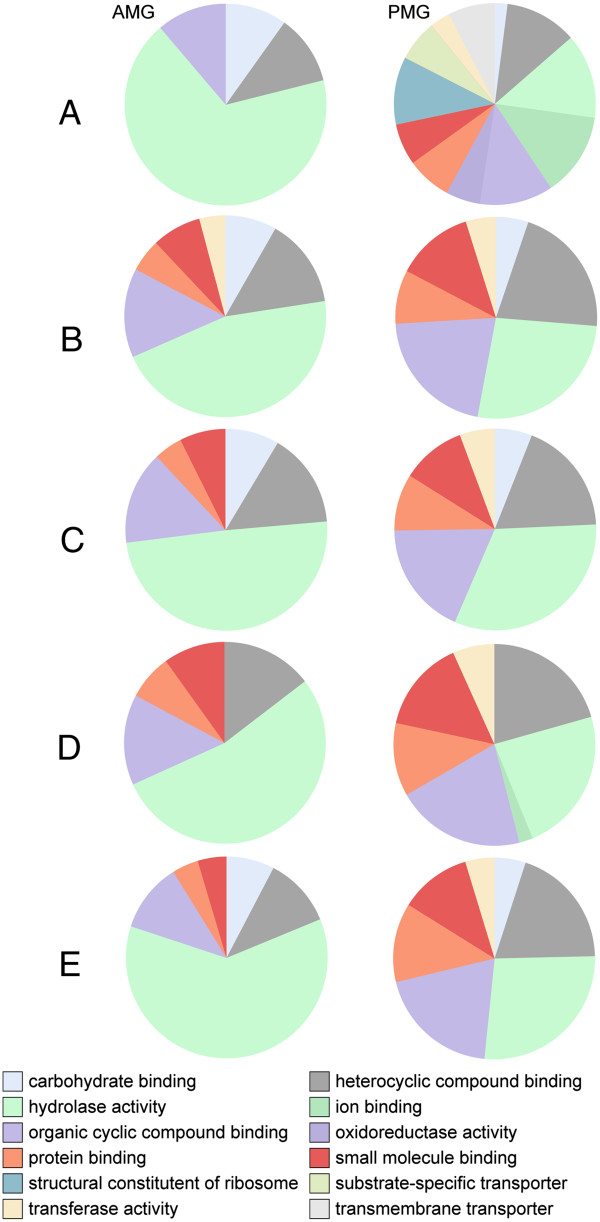
**Comparisons of the GO terms expressed in the anterior and posterior midguts of the Phasmatodea.** The top 500 most expressed transcripts are seen for each of the anterior (left) and posterior (right) midguts. **A)** Aretaon asperrimus. **B)** Extatosoma tiaratum. **C)** Medauroidea extradentata. **D)** Peruphasma schultei. **E)** Ramulus artemis.

**Figure 2:**
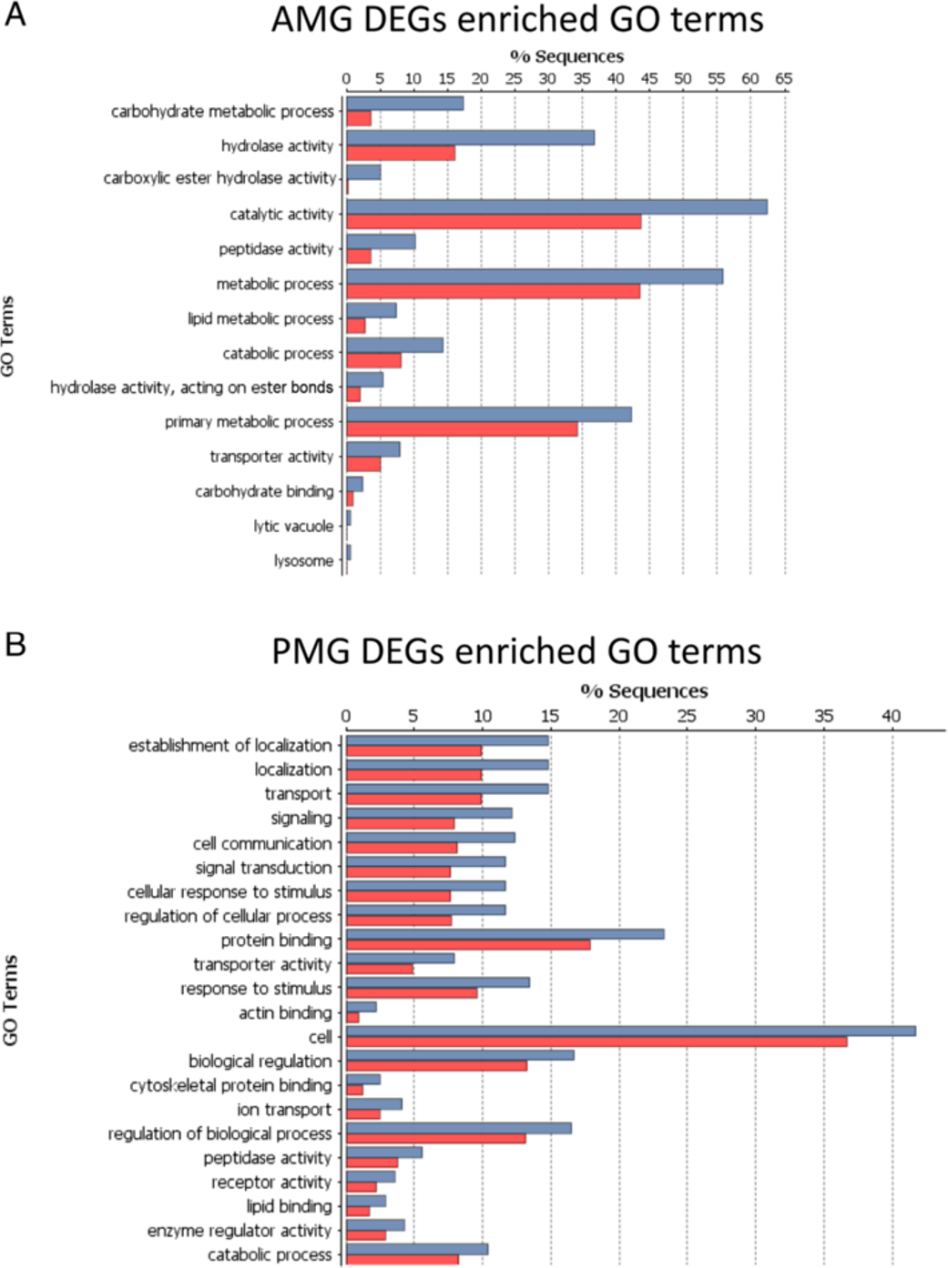
**GO categories enriched for the most differentially expressed genes in each** ***Peruphasma schultei*** **midgut segment.** Values are relative to the overall transcriptome as per Fisher’s exact test. **A)** Anterior midgut (posterior midgut values in red). **B)** Posterior midgut (anterior midgut values in red).

Due to the lack of closely related species with well-annotated genomes, or even consensus as to what is the most closely related order to the Phasmatodea, we used several methods to annotate the assembled transcripts. For all *P. schultei* transcripts and the top 500 most highly expressed transcripts for the other species, we used Blast2GO’s [48] tblastx program to compare each sequence to the NCBI translated nucleotide collection (nr) database, with an expect value threshold of e^-6^. Contigs with highly significant BLAST [49] hits were mapped to the Gene Ontology (GO) database and annotated using Blast2GO with an expect value threshold of e^-6^. InterPro annotations were performed using the Blast2GO remote connection to the InterProEBI server [48]. GO terms were modulated using ANNEX and GOSlim, using the “generic” mapping (goslim_generic.obo) available in Blast2GO (Figure 1). Potential metabolic pathways represented in the transcriptome were identified using the Kyoto Encyclopedia of Genes and Genomes (KEGG) [50] database via Blast2GO (Additional files 1, 2, 3, 4, 5 and 6). Enrichment analysis (Fisher’ s Exact Test via Blast2GO) was used to find enriched GO terms, with term filter value below 0.05, term filter mode “FDR,” and two-tailed test options selected (Figure 2). Annotations were added to those provided by RSEM.

To specifically identify PCWDE-encoding transcripts, we downloaded nucleotide sequences for representative PCWDEs from the NCBI database, selecting known, endogenous insect proteins as well as fungal, bacterial, and protozoan proteins. The query sequences from NCBI were blast-ed against the full transcriptomes after removing low-quality reads, with an expect value threshold of e^-10^. Only transcriptome isoforms that aligned to at least 75% of the representative gene downloaded from NCBI were included in later transcript number analyses.

### Amino acid alignment and phylogenetic analysis

For the putative PCWDEs, the transcript sequences from the phasmids were converted to amino acid sequences using the ExPASy online translation tool [51]. A representative sequence from each isogroup (comp#_c#) was selected based on E-value and Sim mean when compared to known enzymes in the NCBI database. The number of isogroups and isotigs (sequences) within each group is listed in Table 2, and Additional file 7: Table S7 shows these sequence names. Other known protein sequences for these enzymes were collected from the NCBI database from a diversity of organisms including bacteria, fungi, plants, other insects, nematodes, and, when available, protists, chordates, and other invertebrates (Additional file 8: Tables S8, S9, S10 and S11). The amino acid sequences were aligned using MUSCLE [52] and manually curated using Mesquite [53]. Further alignment and production of consensus sequences for clades were done using JalView [54]. These alignments were then searched for the known conserved regions for the active/catalytic sites for each enzyme type, identified using the Catalytic Site Atlas [55], with Blast alignment to confirm their presence in the phasmid transcripts.

**Table 2:** **Number of PCWDE isogroups and isotigs in the phasmid midgut**

	# isogroups (total # isotigs)			
Species	Cellulase	Pectinase	Cellobiase	Beta-1,3-glucanase
Aretaon asperrimus	5 (16)	11 (44)	7 (14)	3 (9)
Extatosoma tiaratum	4 (14)	18 (30)	16 (27)	3 (3)
Medauroidea extradentata	7 (13)	21 (52)	12 (28)	3 (3)
Peruphasma schultei	6 (8)	7 (14)	4 (22)	3 (3)
Ramulus artemis	5 (26)	17 (70)	17 (45)	3 (6)
Sipyloidea sipylus	7 (11)	11 (36)	10 (22)	2 (4)

Data from the full midgut transcriptomes with short sequences removed. Transcripts were identified as PCWDEs based on amino acid alignment to known proteins.

MUSCLE-aligned sequences curated on Mesquite were converted to Phylip format [56]. For neighbor-joining trees, the Phylip program “seqboot” was run to make multiple datasets for bootstrapping, and the results run through “protdist” and “neighbor”, then the trees combined with “consense”. For parsimony trees, the “seqboot” datasets were run through “protpars” and “consense”. For maximum likelihood trees, the MUSCLE-alignment file as uploaded to the CIPRES portal (http://www.phylo.org) [57] and run on RAxML-HPC2 on XSEDE for 1000 bootstrapping runs [58]. For Bayesian analysis, Mr.Bayes 3.2.2 [59] was run with the CIPRES datasets with 500000 generations for bootstrapping. Consensus trees were viewed and prepared for figures using FigTree 1.4.2 [60]. The Maximum Likelihood tables were chosen as the figures for this manuscript.

### Testing for endogenous production of PCWDEs

The possibility existed that some transcripts came from microbial symbionts or contaminants. Table S7 shows which PCWDE encoding isogroups contained poly-adenylation signals, a feature predominantly of eukaryotic mRNA that that, unlike bacterial RNA, will pass in large amount through our cDNA synthesis method, and whose presence is used to suggest endogenicity [61–63]. We also tested the translated transcripts for the presence of eukaryote-specific signal peptides using SignalP 4.1 (http://www.cbs.dtu.dk/services/SignalP/) [64], which is also evidence against bacterial origins for the transcripts. Such methods however cannot differentiate between enzymes produced by protozoan or fungal symbionts and those produced by insects, including insect-produced proteins whose genes were acquired from a eukaryote via horizontal gene transfer as has happened in beetles [23, 30, 65].

To further show that particular PCWDE genes are endogenous to the phasmid genome and not produced by gut symbionts or contaminants, we extracted DNA from non-gut tissue for next generation sequencing. Finding a gene encoding a transcript protein in the genome is a strong and frequently used indicator of endogenicity [27, 66–68]. By using non-alimentary tissue we avoided aspecific amplication of microbial genes and recovered insect DNA alone, as has been done in other insects to show microbe-like genes are endogenously produced [69–71], including the first discovery of endogenously produced cellulase in an insect [72]. We dissected out the brain under sterile conditions from one *P. schultei* individual and extracted DNA using the ChargeSwitch® gDNA Mini Tissue Kit (including the RNAase digestion step). Genomic Illumina libraries for paired-end 100 bp sequencing were then produced using the Illumina Truseq kit and validated using the bioanalyzer 2100. Sequencing was conducted on the Illumina HiSeq 2000, and the data uploaded to the NCBI SRA Database [GenBank:SRP030474]. The numbers of reads generated for the sample are shown in Table 1. We tested if the *P. schultei* cellulase and pectinase genes were endogenous in origin by mapping all genomic reads from the brain tissue back to our *de novo* midgut transcriptome assembly. We then took all the genomic reads that mapped to each PCWDE gut transcriptome gene and blasted them to the entire gut transcriptome to narrow the list of reads down to those that uniquely map to a single PCWDE gene (Table 3).

**Table 3:** ***Peruphasma schultei*** **pectinase and cellulase genomic reads uniquely aligned to transcriptome isotigs**

Pectinases	Genomic reads	Cellulases	Genomic reads
Comp40495_c0_seq1	16	comp22363_c1_seq1	21
Comp54109_c1_seq1	21	comp22404_c0_seq1	23
Comp55819_c1_seq1	40	comp22464_c0_seq1	12
comp55819_c1_seq2	40	comp39876_c0_seq1	20
comp55819_c1_seq3	40	comp55831_c0_seq1	34
comp56173_c8_seq1	40	comp55831_c0_seq2	33
comp56173_c8_seq2	40	comp55831_c0_seq3	40
comp56173_c8_seq3	30	comp57191_c0_seq1	39
comp56173_c8_seq4	39		
comp56691_c1_seq1	41		
comp56691_c1_seq2	20		
comp56826_c1_seq1	40		
comp56826_c2_seq1	40		
comp56826_c2_seq3	40		

These reads are therefore of endogenous genes. Counts to 40 max.

As a final test, we blasted all PCWDE transcripts to the draft genome for *Timema cristinae* (Phasmatodea: Timematidae), which was under development but available at the webpage of the Nosil Lab of Evolutionary Biology at the university of Sheffield, UK (http://nosil-lab.group.shef.ac.uk/?page_id=25). The Timematidae are considered the sister group to all other Phasmatodea [36]. While the *Timema* genome is not guaranteed to have the same genes as the species we examined, finding our transcripts within the *Timema* genome would be strong evidence that the gene is both endogenous in and ancestral to Phasmatodea.

## Results

### Phasmid midgut *de novo*transcriptome assemblies

From the extracted RNA libraries of the pooled AMGs or PMGs of each phasmid species we generated approx 54 million high quality, 100 bp, paired-end sequence reads (Table 1), with the exception of *Sipyloidea sipylus* (Diapheromeridae), from which we were only able to successfully extract RNA from the anterior midguts. *de novo* assembly of each midgut section’s library with Trinity [3] produced ~114-170 thousand transcript contigs per species (Table 1). All reads and the final transcriptome for *Peruphasma schultei* are available under BioProject accession PRJNA221630, and for all other phasmids under PRJNA238833.

### Annotation of the *P. schultei*transcriptomes

Approximately 30323 (22%) of the 135622 transcriptomic sequences had BLAST hits (Additional file 9: Table S9), most of which were homologous to sequences from other insects and arthropods (Additional file 10: Figure S1). More genes were homologous to the red flour beetle *Tribolium castaneum* than to insects from more closely related orders, reflecting the relative dearth of available genetic information from insects in the Polyneoptera clade and the relatively recent sequencing of *Tribolium*. The high percentage of sequences with no blast hits (orthologs) is unsurprising given the lack of an annotated, sequenced Phasmatodea genome. The sequences may have represented noncoding regions, wrongly-assembled contigs, or novel genes whose significance is unknown.

### Differential gene expression across the phasmid midgut

RNA-Seq analysis of *P. schultei* suggested compartmentalization of gut function (Figure 2), as found in other insects [24] and plant cell wall consuming organisms. Additional files 11 and 12 list all differentially expressed genes (DEGs) between the *P. schultei* midgut sections, defined as genes with a Posterior Probability of Differential Expression (PPDE) >0.95 [47]. We also defined genes as being highly expressed if their expression levels were 10× higher than the mean for that midgut segment. Over 4000 genes were differentially expressed in each gut section, with 2318 genes expressed only in the AMG and 1309 expressed only in the PMG. We found 318 highly expressed genes for the AMG and 648 for the PMG (Additional files 13 and 14).

Analysis of the most highly expressed sequences in each midgut section for all species further suggested compartmentalization of digestion, or at least enzyme gene expression. All species showed similar GO category profiles for each midgut section (Figure 1), with nearly 50% reduction in hydrolase gene expression in the PMG relative to the AMG. Enzyme-encoding transcripts that break down polymers at internal sites, such as serine proteases, lipases, and PCWDEs [73] were more abundant in the AMG, as were carboxylesterases transcripts and sugar hydrolases. Transcripts abundant in the PMG encoded enzymes that break down dimers and monomers, such as dipeptidases, phospholipases, and trehalase, as well as some cell membrane receptor proteins and cytochrome P450s.

### Phasmatodea midgut PCWDEs

Among the sugar hydrolases were several isogroups of PCWDEs including cellulases in the GH9 family and cellobiase, which together can digest cellulose polymers completely into sugar, and the pectinase endopolygalacturonase. We also found transcripts encoding beta-1,3-glucanase (EC:3.2.1.39), a polysaccharide-degrading enzyme family known mostly from Lepidoptera larval midguts and expressed in response to feeding on a diet containing bacteria [74]. These four enzymes (cellulases, cellobiases, pectinases, and beta-1,3-glucanases) were used in the manual annotations.

### Amino acid alignment and phylogenetic analysis

**Figure 3:**
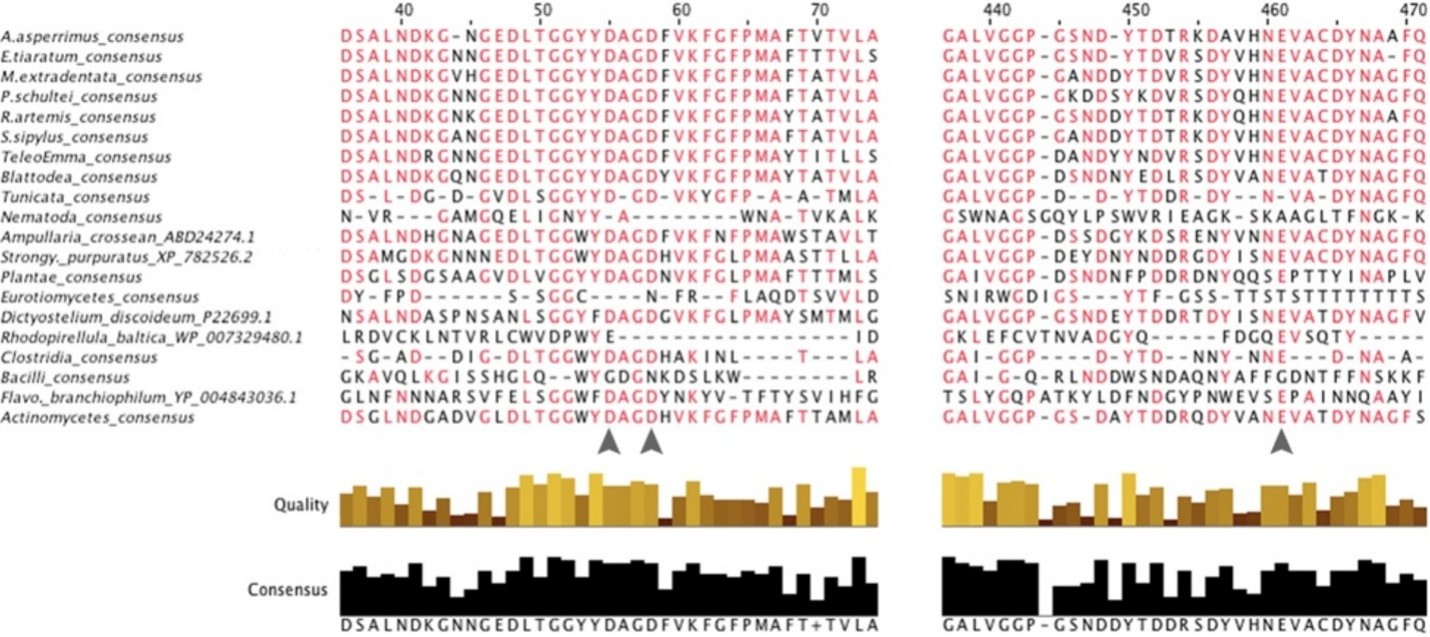
**Sections of cellulase amino acid sequence alignments containing conserved/active site residues.** Catalytic sites are identified by the grey arrows [75, 76]. List of Phasmatodea sequences in Additional file 7, and all others in Additional file 7. Similar sequences from groups of related species were combined into consensus sequences. Red letters show identity with the overall consensus sequence based on conservation of physiochemical properties. Quality is the inverse likelihood of observing mutations based on the BLOSUM62 matrix [54]. *Strongylocentrotus purpuratus* and *Flavobacterium branchiophilum* are abbreviated.

The phasmid cellulases aligned most closely with other Polyneopteran cellulases — including the known, active, endogenous cellulases isolated from the phasmids *Eurycantha calcarata* and *Entoria okinawaensis* [27] — as well as those of other invertebrates, tunicates, plants, and actinomycete bacteria, but not with nematode or beetle cellulases. Phasmid endoglucanases are of the GH9 family thought to be ancestral to all animal life [21], as opposed to the GH5, 45, or 48 cellulases found in nematodes and beetles [23]. The phasmid transcripts either themselves included or were homologous to transcripts including the known active sites invariant in GH9 cellulases, based on work on *Thermobifida/Thermomonospora fusca* (PDB: 1js4) [56, 57]: namely two conserved Asp’s (D55, D58) functioning in catalytic base activity and a Glu residue (E461) that functions as the catalytic acid (Figure 3). Phylogenetic analysis could not resolve domain or phylum-level relationships among the sequences tested (bootstrap values <10). Every cellulase transcript we isolated was homologous to two sequences from the *Timema* genome.

**Figure 4:**
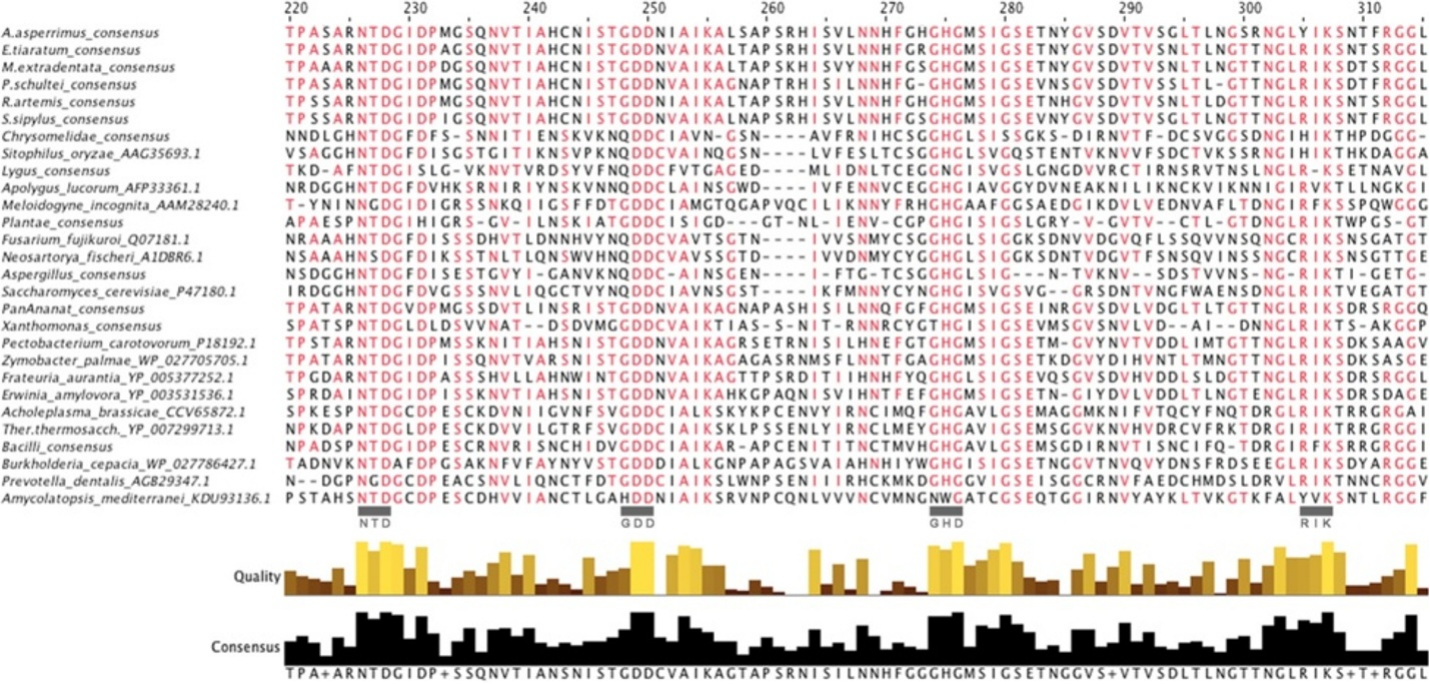
**Section of pectinase amino acid sequence alignments containing conserved/active site residues.** See caption to Figure 3. Catalytic residues identified with grey bars [77]. *Thermoanaerobacterium thermosaccharolyticum* is abbreviated*.*

**Figure 5:**
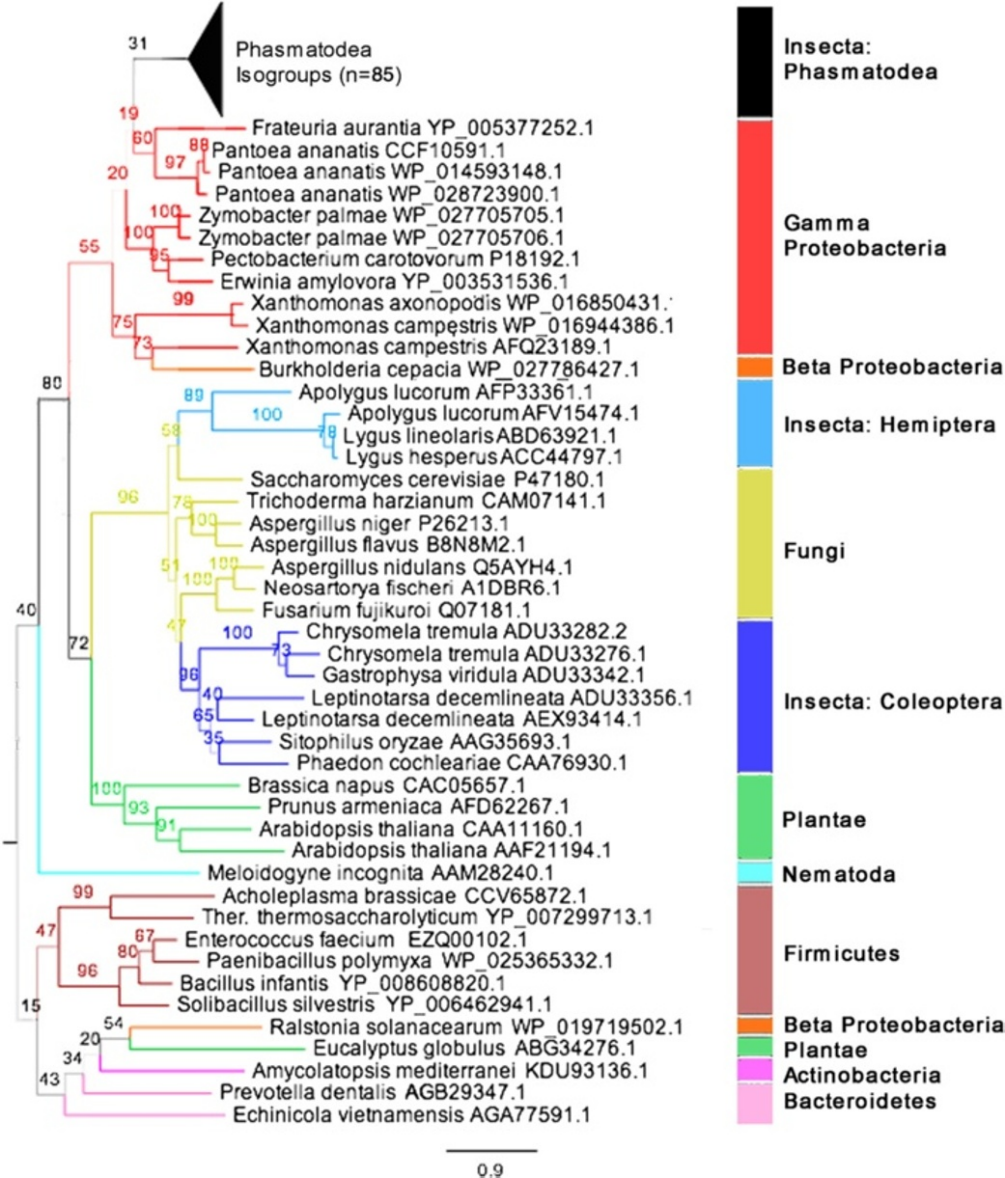
**Maximum likelihood tree for the pectinases.** Phylogenetic analysis of amino acid sequences made with RAxML-HPC2 on the XSEDE system [57]. Numbers are bootstrap values (1000 runs). Branch widths based on bootstrap value, branch colors based on clade. Branch lengths based on the mean number of nucleotide substitutions per site (Scale Bar =0.9). All Phasmatodea sequences (Additional file 7) were a monophyletic group among the gamma proteobacteria. *Thermoanaerobacterium thermosaccharolyticum* is abbreviated*.*

The phasmid pectinase sequences aligned most closely with those of gamma proteobacteria, rather than other insects or eukaryotes. The alignment also showed Hemipteran and beetle pectinases as most similar to fungal pectinases, the latter homology already noted in the literature [30, 65]. Nearly all phasmid pectinase enzymes contained the four conserved regions of the catalytic sites, based on work on *Erwinia carotovora* (PDB: 1bhe) [77]: Asn226-Thr227-Asp228, Gly248-Asp249-Asp250, Gly274-His275-Gly276, and Arg305-Ile306-Lys307 (Figure 4). An exception is the transcripts from *A. asperrimus*, whose Arg residue is replaced with a Tyrosine (Y305). Phylogenetic analysis suggested the phasmid polygalacturonases are a monophyletic group within those of the gamma proteobacteria (Figure 5).

The possibility exists that the pectinase transcripts came from gut bacteria or contaminants rather than phasmid genes. However, many of the pectinase transcripts had poly-A tails, as did those of other PCWDEs (†in Table S7), and all the PCWDE transcripts that were not truncated at the 5’ end had eukaryotic-specific signal peptides. This includes transcripts that contained complete open reading frames (*in Table S7) as well as those that were 3’ truncated. Each pectinase-encoding contig also had multiple (in most cases very many) matching genomic reads from brain tissue that uniquely aligned to them (Table 3). The same matching genomic reads could be found for cellulases, which have been demonstrated to be endogenously produced in phasmids [27] and are endogenously produced in many other insects [17] and metazoans [21]. However, none of the pectinase transcripts had homologues in the *Timema* genome.

**Figure 6:**
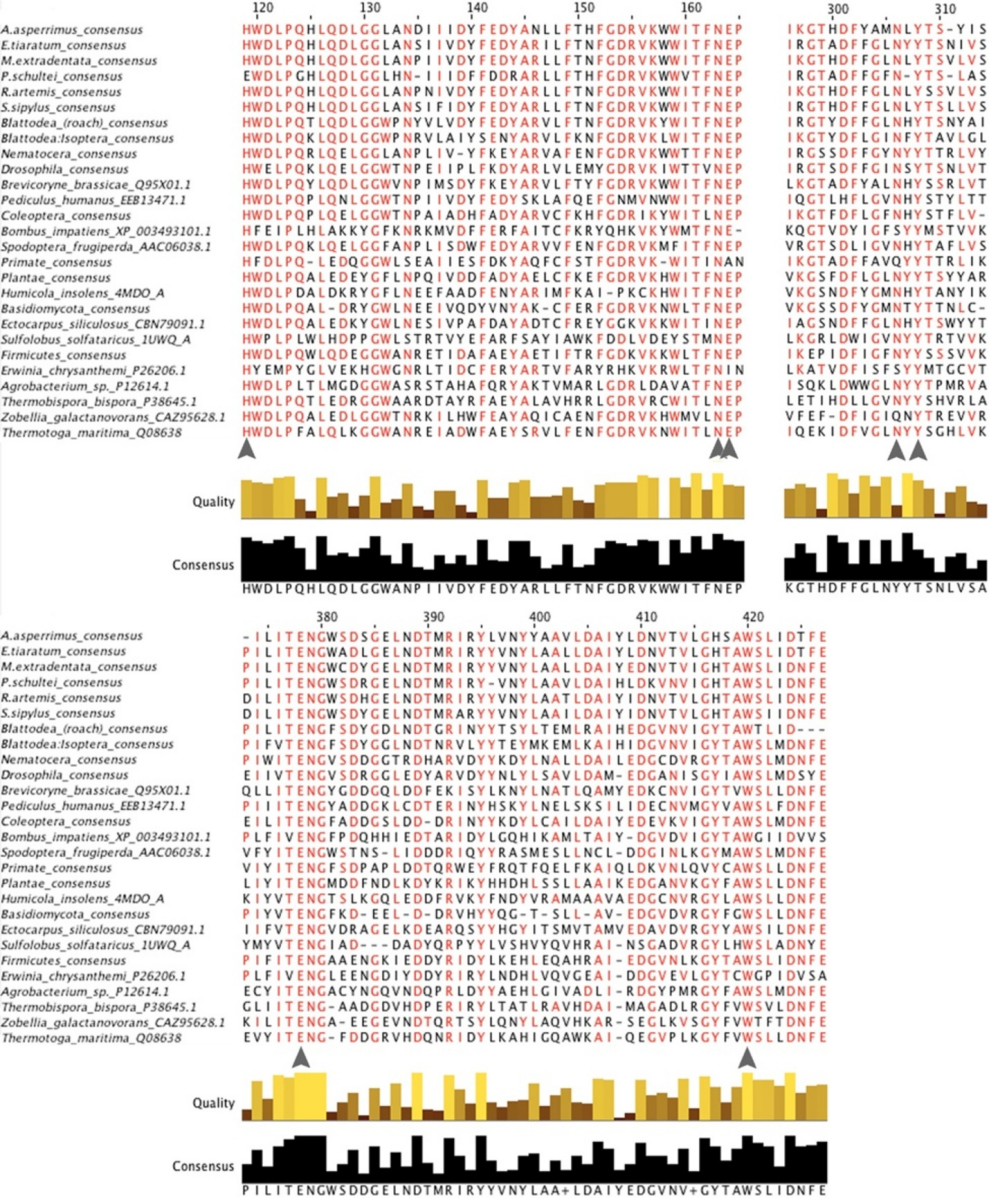
**Sections of cellobiase amino acid sequence alignments containing conserved/active site residues.** See caption to Figure 3. Catalytic and conserved residues identified with grey arrows [78].

**Figure 7:**
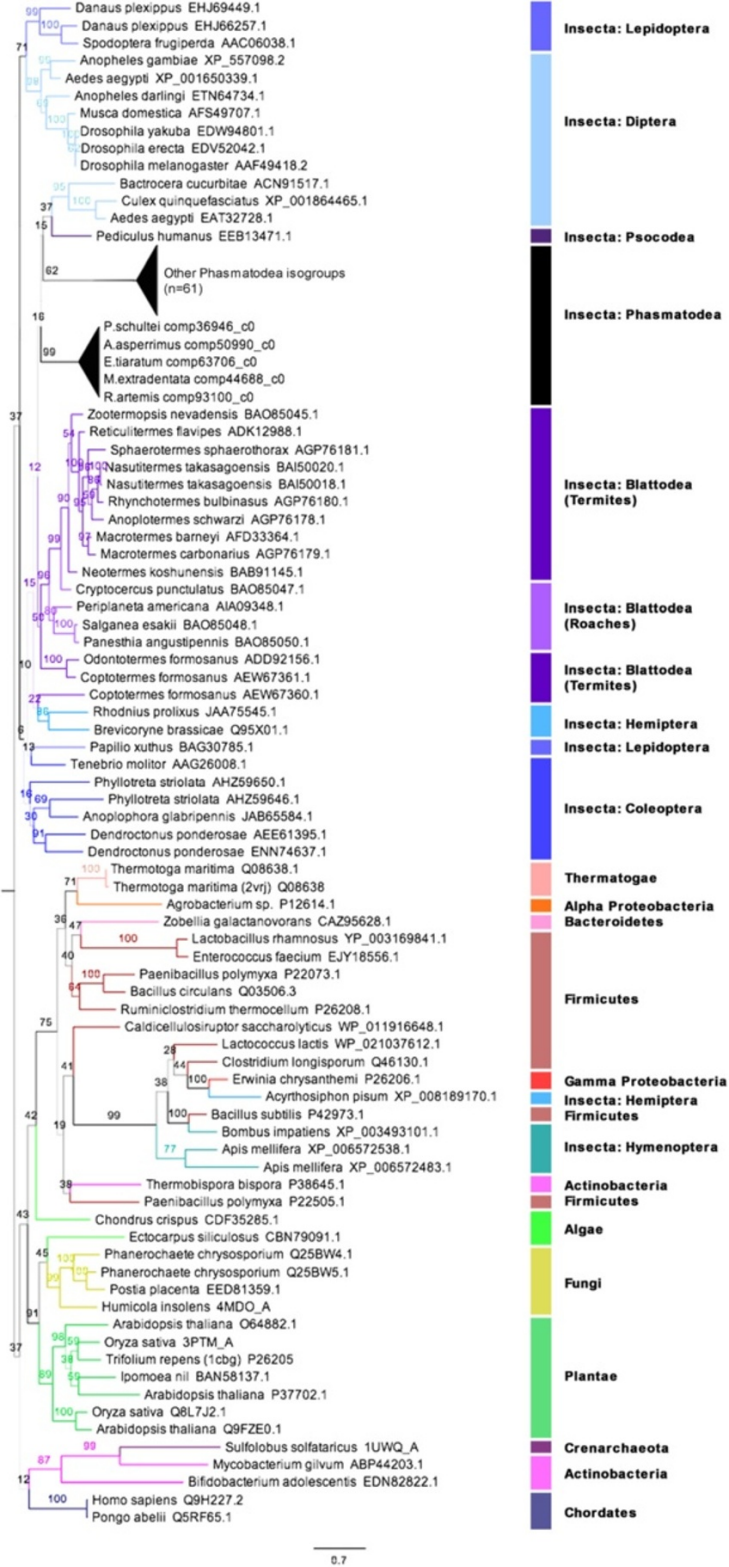
**Maximum likelihood tree for the beta-glucosidases.** Phylogenetic analysis of amino acid sequences made with RAxML-HPC2 on the XSEDE system [57]. Numbers are bootstrap values (1000 runs). Branch widths based on bootstrap value, branch colors based on clade. Branch lengths based on the mean number of nucleotide substitutions per site (Scale Bar =0.7). The Phasmatodea sequences (Additional file 7) formed two strongly supported monophyletic group with weak relationships to other insect groups.

The phasmid beta-glucosidases/cellobiases were in the GH1 family and aligned most with those of other insects. The phasmid transcripts mostly had the conserved residues of beta-glucosidases, including the catalytic sites, based on work with white clover, *Trifolium repens* (PDB: 1cbg) [78]: Arg75, His119, Asn163, Glu164, Asn306, Tyr308, Glu378, and Trp420 (Figure 6). Phylogenetic analysis suggested the phasmid cellobiases are nearly all monophyletic, except a strongly-supported clade consisting of one isogroup from each species but *S. sipylus* (Figure 7). Analysis could not determine interclass relationships among insect beta-glucosidases, but suggested the enzyme existed in the common ancestor of the Insecta. Every beta-glucosidase transcript we isolated was homologous to four sequences from the *Timema* genome.

**Figure 8:**
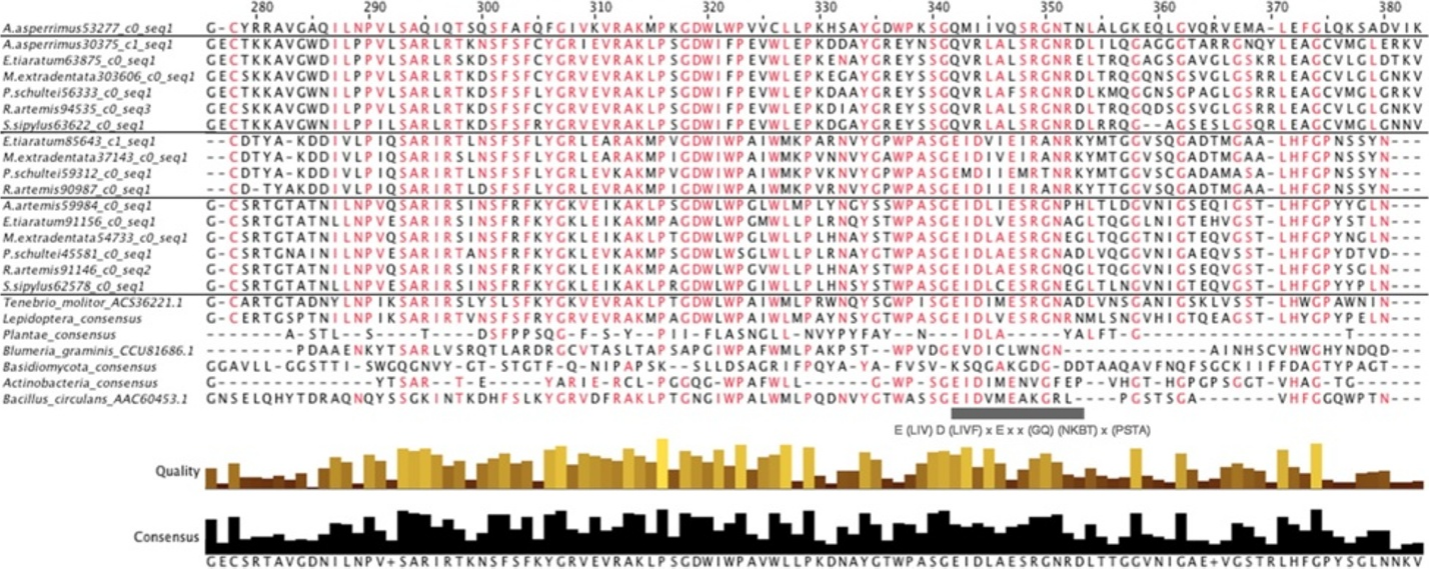
**Sections of beta-1,3-glucanase amino acid sequence alignments containing conserved/active site residues.** See caption to Figure 3. Conserved, twelve amino acid region identified with grey bar [79]. The four groups of Phasmatodea gene are separated by black lines.

**Figure 9:**
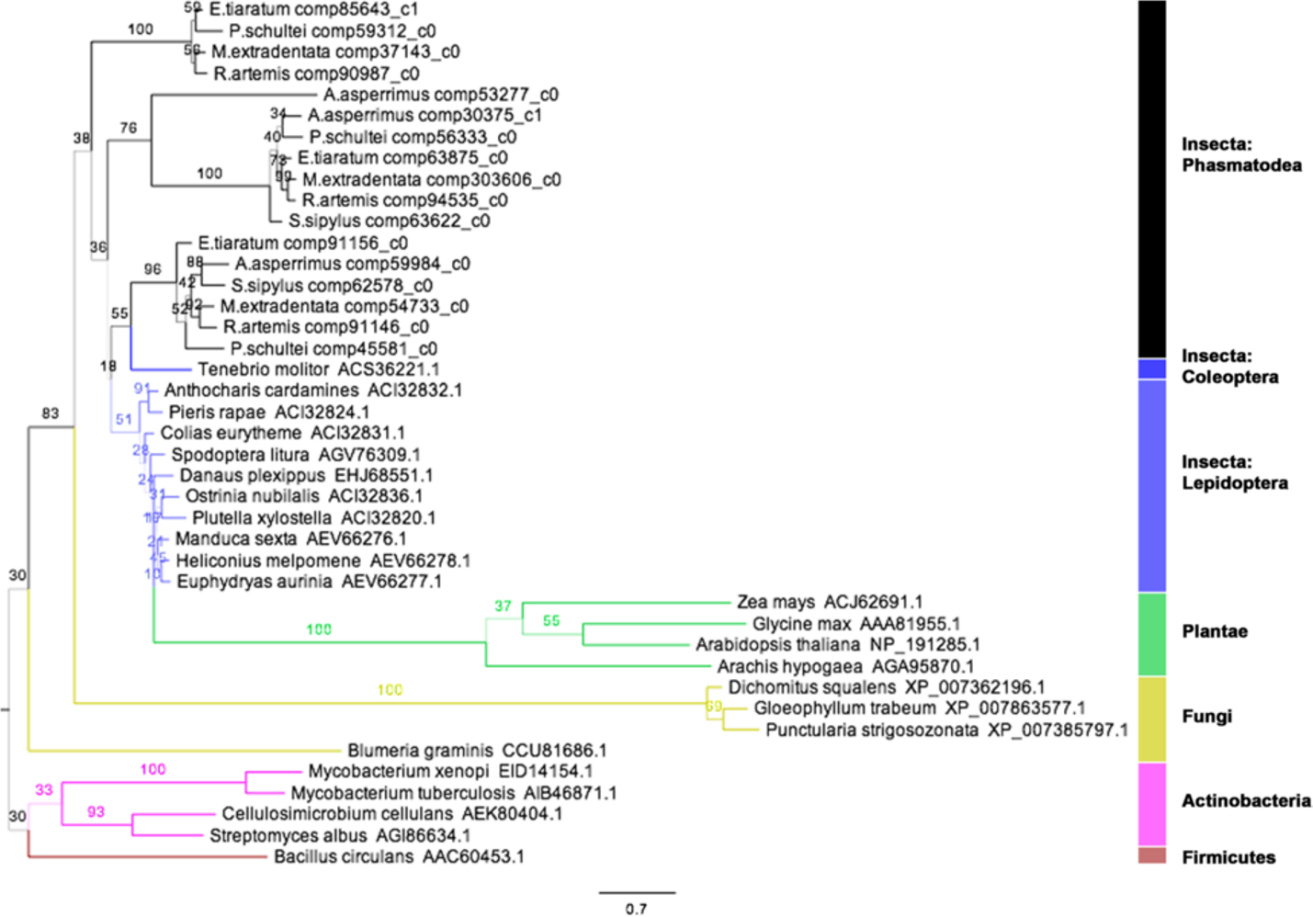
**Maximum likelihood tree for the beta-1,3-glucanases.** Phylogenetic analysis of amino acid sequences made with RAxML-HPC2 on the XSEDE system [57]. Numbers are bootstrap values (1000 runs). Branch widths based on bootstrap value, branch colors based on clade. Branch lengths based on the mean number of nucleotide substitutions per site (Scale Bar =0.7). The Phasmatodea sequences formed four strongly supported groups.

The phasmid beta-1,3-glucanases were in the GH16 family and aligned most closely with other insect enzymes (Figure 8), however this is a relatively recently described enzyme with few recorded sequences in the literature or NCBI database. This is the first known record of endogenous beta-1,3-glucanase in the Polyneoptera. The phasmid beta-1,3-glucanases could be divided into four clear, monophyletic groups (Figure 9) with no more than one representative isogroup per each of the six phasmid species. Each group differed in their homology with the known consensus pattern for catalytically active beta-1,3-glucanases based on work on *Bacillus licheniformis* [79]: E-[LIV]-D-[LIVF]-x(0,1)-E-x(2)-[GQ]-[KRNF]-x-[PSTA] (Figure 8: 342–353). One group of six sequences had 11/12 amino acids conserved, a group of four had 10/12, another group of six had 8/12, and the final group consisting of one *A. asperrimus* sequence had 6/12. The last amino acid in the *Bacillus* region was not conserved among any phasmids, nor is it conserved among the Lepidoptera sequences, which were also 11/12, or many other organism sequences sampled. Every beta-1,3-glucanase transcript we isolated had six to eight homologues in the *Timema* genome.

## Discussion

Using high coverage sequencing of RNA expressed in their midguts, we were able to produce high quality transcriptomes of several Phasmatodea species. This new data doubles the genera of phasmids with publicly available genetic resources on the NCBI databases, increasing the amount of annotated genes available for future work not only on Phasmatodea, but also on the Polyneoptera in general. Covering six species in four families, while drawing from the draft genome of a seventh species in a fifth family, the data suggests the differential expression and enzyme gene diversity of the phasmid midgut sections is mostly conserved throughout the order. Our findings will serve as a reference set for studying phasmid digestion and a jumping point for future proteomic and biochemical assays. The abundance of PCWDE isogroups in phasmids is relatively high, and the diversity of PCWDE types is comparable to those in certain leaf beetles (Chrysomelidae) like *Phaedon cochleariae* [69, 71] or wood-boring beetles (Cerambycidae) like *Anoplophora glabripennis* [80]. The current record is likely *Diabrotica virgifera virgifera* (Chrysomelidae) with seventy-eight genes putatively encoding proteins from the same four enzyme classes studied here [55]. As Phasmatodea and Chrysomelidae are among the few insect groups to be exclusively folivorous, a possible correlation exists between that dietary niche and a diverse PCWDE complement.

For *de novo* transcriptomes, assemblers such as Trinity often cannot differentiate between homologous genes and isoforms or allelic variants of the same gene. They can potentially overestimate the number of isotigs (single or groups of contigs that should each constitute one splice variant) within an isogroup (all isotigs for one gene, identified by Trinity’s output as comp#_c#) [81]. Combined with relatively low genetic resource availability for closely related insects and relatively high representation of species like the aforementioned beetles, we cannot be certain whether phasmids express more or fewer PCWDEs than the average herbivorous insect. In addition to using programs like RSEM designed to reduce such errors [46], comparing the number of reads mapping to a locus on the genome can be used to infer the true isoform number and account for inflation [82]. That most *P. schultei* transcriptome sequences (contigs) had more matching genome reads (Table 3) than their corresponding isogroup has members (Table 2) suggests that our contig numbers represent true isoforms within each isogroup, rather than an overestimation due to mis-assembly [82, 83]. These phasmid isoforms may reflect multiple gene copies or alternatively spliced genes, either case suggesting a diverse complement of proteins working together to fully digest multiple varieties of carbohydrate polymer. a highly derived genetic capacity for plant cell wall breakdown [84]. However, because some isotigs were truncated at the 3’ or 5’ end, the possibility exists that certain transcripts represent different ends of a single gene. Future work using RACE-PCR from primers based on the transcripts identified here would produce full-length cDNA sequences that will determine which transcripts represent unique genes and which are fragments, Such genes could then be expressed into insect cell cultures for use in downstream enzymatic activity assays [85].

Previous research has confirmed that the endogenous cellulase genes we demonstrated are most highly expressed in the anterior midgut are also most highly active in the anterior midgut [27], making it the site of both cellulase translation and action. The physical structure of the AMG supports this hypothesis: the pleating and folding serves to greatly increase the available surface area of the AMG while slowing down the transit speed of food, increasing the amount of time and space available for cellulase enzymes to hydrolyze ingested plant material. Cellulase activity falls to nearly nothing in the PMG, tracking with cellulase gene expression. If we extend the results of cellulases to those of the other PCWDEs, then we hypothesize that phasmid digestive enzymes are active in the same region of the gut where they are expressed, making the pleated AMG the site of primary plant cell wall and polymer digestion and the PMG the site of secondary digestion of smaller oligomers at most.

We also hypothesize that phasmids can fully digest cellulose into glucose, as they have the two enzymesnecessary to do so, and also actively degrade pectin into galacturonic acid. Such digestive abilities could explain how phasmids survive on otherwise uncommon, obligately folivorous diets: by fully breaking down plant cell walls into assimilatable nutrients rather than just degrading the walls to access the nutrient-rich cytoplasm within. As transcriptomics only demonstrates gene expression, not translation or activity, these hypotheses cannot be confirmed with this data alone. However, the fact that phasmids have active cellulases [27] and the presence of the relevant catalytic residues on the phasmid cellulase, pectinase, and cellobiase transcripts (Figures 3,4,6) and some beta-1,3-glucanase transcripts (Figure 8) suggests the transcripts code for functional enzymes, supporting the hypothesis that these enzymes are indeed actively degrading plant cell walls. We have thus provided the necessary preliminary work justifying biochemical and proteomic assays into cellobiase, pectinase, and beta-glucanase activity in the phasmid gut.

Phasmid pectinases are all endopolygalacturonases in the GH28 group, known in insects only from the beetles and the Hemiptera, but they show homology and align to those from gamma proteobacteria (Figure 4). Pectinase genes were also absent in the *Timema* genome. Our pectinase transcripts may have come from a bacterial symbiont. The successful mapping of all *P. schultei* pectinase and cellulase transcripts to genes in the *P. schultei* genome, the presence of eukaryote-specific poly-A tails and signal peptides on phasmid transcripts with complete open reading frames, previous studies with *P. schultei* and *R. artemis* suggesting their digestion is symbiont independent [33], and the absence of characteristic paunches for microbial fermentation [37], all tentatively suggest these pectinases are encoded in the phasmids own genome and not produced by gut microbes. However, the possibility remains that the samples were contaminated by a non-symbiotic microbe: either a rare bacteria that poly-adenylates its RNA or a fungal symbiont that acquired a bacterial gene via horizontal transfer.

A more parsimonious hypothesis is that the phasmid pectinase gene was acquired through horizontal transfer from a bacterial ancestor, much as the beetle pectinases are thought to have been acquired though horizontal transfer from an Ascomycete fungus [23, 30, 65], or as leaf beetle xylanases may have also transferred from a gamma proteobacteria [69]. The absence of the genes in *Timema* would suggest either that the transfer event occurred after the split between the Timematidae and the other Phasmatodea, or that the pectinase genes are ancestral to both and lost in *Timema*. Lastly, the similarity between phasmid and bacterial sequences could simply be an artefact of the over-representation of microbial and dearth of animal pectinases in the NCBI database at this time [86]. Using long range or RACE PCR to clone entire genes from phasmid genomic DNA and get introns would conclusively demonstrate whether or not the pectinases are endogenously produced or not, and such work on all six species studied here is underway.

Whether pectinase genes exist in other Polyneoptera remains to be seen, but would help determine when the horizontal transfer event could have occurred. Failure to find endogenous pectinases in closely related insects would mean the transfer occurred in an early Phasmatodea ancestor: a development that would have expanded the digestive abilities of the order and may have played a significant role in their evolution of obligate folivory. PCWDE diversity could also be correlated to the development of the longer and larger body sizes of the Euphasmatodea. Broader, multi-phyla, phylogenetic analysis for pectinolytic enzyme genes in the Animalia can answer this question [35].

Phasmid cellobiases are GH1, as are those of other insects who produce them endogenously like the higher termites [87]. Such species can break down cellobiose independently, unlike the lower termites that have symbiotic microorganisms to produce their cellobiases for them. The beta-1,3-glucanases are GH16, similar to those found in Lepidoptera [74], however the prevalence of this recently described gene in animals has not been sufficiently examined. A greater sampling of this gene's presence in other animal, fungal, and bacterial species, as well as biochemical studies to determine the conserved catalytic residues for the protein, are needed before ordinal-level hypotheses can be made for the enzyme's evolutionary history. So far, the animal enzymes appear most closely related, and we hypothesize that at least three distinct beta-1,3-glucanase gene families existed in an early Phasmatodea ancestor, including the ancestor of the Timematidae.

Our work promotes phasmids as a potentially high-value source of novel PCWDEs for biotechnological applications [70]. Cellulases and pectinases are highly sought after by the biofuel industry to degrade feedstock into the monomers later converted into fuel, or to improve the flow rate of the material by reducing the amount of solid matter [39]. Pectinases are also used in the production of coffee, tea, and juice, and in waste-water treatment [88]. Phasmid PCWDEs could be introduced into bacteria or fungi like *Trichoderma reesei*, for industrial-scale enzyme production or direct use in bioreactors for wastewater treatment or biofuel production [89].

Our *de novo* midgut transcriptomes enabled us to survey all expressed PCWDEs of the Phasmatodea at once and identify conserved catalytic domains, justifying downstream translation and activity level analyses. A benefit of this is system is the increased speed and efficiency compared to the converse [90]: running chemical assays to identify enzyme activity, using proteomics to identify the amino acid sequence of isolated enzymes, and working backwards from there to design a primer for the enzyme-encoding gene and hope it exists within the target organism’s genome itself and not a symbiont or contaminant [91]. Another benefit is that transcriptomics can reveal genes useful for phylogenetic analysis but that are not translated or whose proteins are modified post-translation such that the standard biochemical tests do not detect their function. An associated drawback is that expressed genes are not necessarily translated into active proteins, nor are they necessarily active at the site of expression [92]. However, when a reference genome is not available, a transcriptome can provide large sets of potential genes for study and, combined with genomic data, can determine whether or not they are endogenous to the target organism, which cannot be determined by homology alone. *de novo* transcriptome assembly combined with RNA-Seq is a powerful tool for suggesting putative functions for unknown tissues in understudied organisms and directions for future study.

## Conclusions

The folivorous Phasmatodea are an ideal system to study the evolution of obligate herbivory, yet a paucity of genetic resources and poorly understood basic biology impede such work. Using RNA-Seq, we demonstrated a diversity of plant cell wall degrading enzymes expressed differentially in the anterior section of the phasmid midgut. Of these, the cellulases, cellobiases, and beta-1,3-glucanases are likely all encoded in the insect’s own genome, as are the pectinases, though we could not definitively rule out a microbial source for the latter. Such an abundance of endogenous enzymes was not expected from the Polyneoptera, raising important questions on their evolutionary history. The efficiency by which our *de novo* transcriptomes generated new genomic resources and hypotheses for future research on Polyneopteran digestion demonstrate the power of such methods to analyze organisms lacking sequenced genomes. Our findings strongly encourage expanding the searches for PCWDEs, most notably the pectinases and beta-1,3-glucanases, into other, lower Polyneopteran insects.

### Availability of supporting data

All reads and sequence files described in the manuscript are available under BioProject accessions PRJNA221630 for *P. schultei* and PRJNA238833 for the other phasmids.

## Electronic supplementary material

Additional file 1: Table S1: KEGG Table for the most highly expressed genes of the *Aretaon asperrimus* midgut. Top 500 most highly expressed genes in each of the anterior midgut (AMG) and posterior midgut (PMG) used. (XLS 27 KB)

Additional file 2: Table S2: KEGG Table for the most highly expressed genes of the *Extatosoma tiaratum* midgut. Top 500 most highly expressed genes in each of the anterior midgut (AMG) and posterior midgut (PMG) used. (XLS 30 KB)

Additional file 3: Table S3: KEGG Table for the most highly expressed genes of the *Medauroidea extradentata* midgut. Top 500 most highly expressed genes in each of the anterior midgut (AMG) and posterior midgut (PMG) used. (XLS 25 KB)

Additional file 4: Table S4: KEGG Table for the most highly expressed genes of the *Peruphasma schultei* midgut. Top 500 most highly expressed genes in each of the anterior midgut (AMG) and posterior midgut (PMG) used. (XLS 42 KB)

Additional file 5: Table S5: KEGG Table for the most highly expressed genes of the *Ramulus artemis* midgut. Top 500 most highly expressed genes in each of the anterior midgut (AMG) and posterior midgut (PMG) used. (XLS 24 KB)

Additional file 6: Table S6: KEGG Table for the most highly expressed genes of the *Sipyloidea sipylus* midgut. Top 500 most highly expressed genes in each of the anterior midgut (AMG) only. (XLS 11 KB)

Additional file 7: Table S7: Representative isotigs (sequences) for each PCWDE isogroup for the phasmatodea. Data is from the full midgut transcriptomes with short sequences removed. Transcripts were identified as PCWDEs based on amino acid alignment to known proteins from the NCBI database. * = The sequence contained a complete open-reading frame. Other sequences could have been truncated at the 5’ or 3’ or both. † = The sequence or another in that isogroup (_c#) had a poly-A tail. (XLS 22 KB)

Additional file 8: Table S8: – Species and NCBI Accession No's for cellulase (Beta-1,4-endoglucanase) proteins compared to phasmid proteins. Sequences with *were not included in the alignment figure (Figure 4) due to poor or absent overlap with other sequences at that region. **Table S9.** – Species and NCBI Accession No's for pectinase (polygalacturonase) proteins compared to phasmid proteins. Sequences with *were not included in the alignment figure (Figure 5) due to poor or absent overlap with other sequences at that region. **Table S10.** – Species and NCBI Accession No's for cellobiase (beta-glucosidase) proteins compared to phasmid proteins. Sequences with *were not included in the alignment figure (Figure 7) due to poor or absent overlap with other sequences at that region. **Table S11.** – Species and NCBI Accession No's for beta-1,3-glucanase proteins compared to phasmid proteins. Sequences with *were not included in the alignment figure Figure 9) due to poor or absent overlap with other sequences at that region. (XLS 60 KB)

Additional file 9: Table S9: Number of *Peruphasma schultei* midgut transcriptome sequences with successful Blast search, mapping, and annotation. (TXT 163 bytes)

Additional file 10: Figure S1: Species distribution for top-hit Blast results of *P. schultei* midgut transcriptome. (PDF 4 KB)

Additional file 11: Table S12: The most differentially expressed genes (DEGs) in the *P. schultei* AMG. Includes genes found in both or only one tissue type. Means measured in RPKM. PPDE = Posterior Probability of Differential Expression. Annotations made with Blast2GO. (XLS 3 MB)

Additional file 12: Table S13: The most differentially expressed genes (DEGs) in the *P. schultei* PMG. Includes genes found in both or only one tissue type. Means measured in RPKM. PPDE = Posterior Probability of Differential Expression. Annotations made with Blast2GO. (XLS 3 MB)

Additional file 13: Table S14: The most highly expressed genes of the *P. schultei* AMG. Identified as genes with expression levels ten times greater than the mean for that section. Means measured in RPKM. Annotations made with Blast2GO. (XLS 3 MB)

Additional file 14: Table S15: The most highly expressed genes of the *P. schultei* PMG. Identified as genes with expression levels ten times greater than the mean for that section. Means measured in RPKM. Annotations made with Blast2GO. (XLS 3 MB)

## Abbreviations

AMG: Anterior midgut
CIPRES: Cyberinfrastructure for phylogenetic research
DEG: Differentially expressed genes
EC: Enzyme commission
GH: Glycoside hydrolase
GO: Gene ontology
HEG: Highly expressed gene
KEGG: Kyoto encyclopedia of genes and genomes
NCBI: National center for biotechnology information
PCWDE: Plant cell wall degrading enzyme
PDB: Protein data bank
PMG: Posterior midgut
PPDE: Posterior probability of differential expression
RPKM: Reads per kilobase per million
RSEM: RNA-Seq by expectation maximization
SRA: Sequence read archive
XSEDE: Extreme science and engineering discovery environment.
