# Supplementary figures and images for: Differential expression of endogenous plant cell wall degrading enzyme genes in the stick insect (Phasmatodea) midgut
Source: BMC Genomics. 2014 Oct 21;15(1):917. doi: 10.1186/1471-2164-15-917 (PMC4221708; doi:10.1186/1471-2164-15-917)

# Top-Hit species distribution

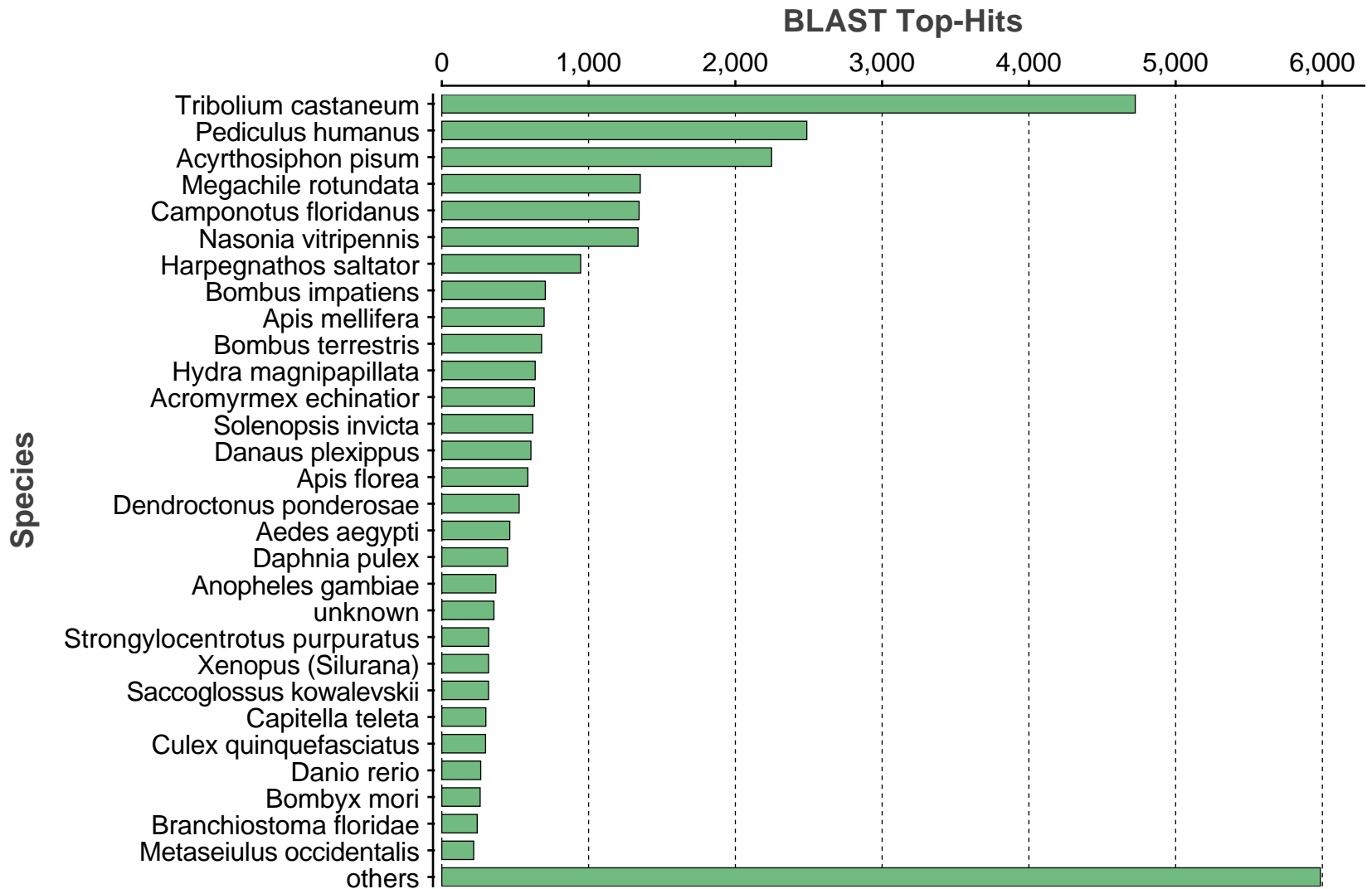

Supplement: Supplementary file 10 — Additional file 10: Figure S1: Species distribution for top-hit Blast results of P. schultei midgut transcriptome. (PDF 4 KB) [file 12864_2014_6620_MOESM10_ESM.pdf]
